# A lifespan approach to understanding reserve and resilience

**DOI:** 10.1002/alz.087229

**Published:** 2025-01-09

**Authors:** Kristine B Walhovd, Anders M Fjell, Didac Vidal‐Piñeiro, Sarah‐Naomi James, Naiara Demnitz, Tessa Harrison, Øystein Sørensen

**Affiliations:** ^1^ Computational Radiology and Artificial Intelligence, Oslo University Hospital, Oslo Norway; ^2^ LCBC, University of Oslo, Oslo Norway; ^3^ MRC Unit for Lifelong Health and Ageing at UCL, London United Kingdom; ^4^ Danish Research Centre for Magnetic Resonance, Hvidovre Denmark; ^5^ University of California at Berkeley, Berkeley, CA USA

## Abstract

**Background:**

That some individuals fall below a functional threshold sooner than others, can be ascribed to differences in “brain maintenance”, slope of change, or variation in previous level, intercept. Intercept differences may be captured in the concept “brain reserve”. Searching for factors that modify outcomes, we need to distinguish how such factors associate with differences in level versus slope of brain and cognition. This necessitates longitudinal data from multiple cohorts since associations can be small and represent different conditions and covariates across samples. Unfortunately, data from longitudinal cohorts are often not readily sharable.

**Method:**

The results of meta‐and mega‐analytic techniques for age‐relationships of neuroimaging and other measures can be compared using data from the Lifebrain consortium (Walhovd et al. Eur Psychiatry. 2018 Jan;47:76‐87). Meta‐GAM, a method for meta‐analysis of generalized additive models (Sørensen et al. NeuroImage 2021;224:117416), yields valid results for modeling non‐linear relationships characteristic of lifespan trajectories. Using meta‐GAM requires less harmonization of measures and enables well‐powered cross‐sample analysis by estimating the relationships of interest separately in each data location without sharing the raw data. Meta‐GAM will be used to test the associations of modifiable and non‐modifiable factors with intercepts and changes in brain and cognition across multiple cohorts. All cohorts will have brain MRI and measures of cognitive function, in addition to factors of interest depending on cohort, such as early life (e.g. birth weight, parental SES), genetics and adult SES and lifestyle variables (e.g. physical activity, dietary pattern).

**Results:**

Preliminary results indicate that individual differences in the level of brain and cognition may appear more stable, larger, and more pervasive than differences in change across the lifespan (Walhovd et al. TICS,2023;27:10). New analyses will focus on the relative strength of associations between potentially modifiable factors, intercept and change of cortical volume and general cognitive ability.

**Conclusion:**

This initiative will allow collaboration and synergies in large‐scale, multi‐sample analyses of cohorts that cannot be shared due to regulatory, privacy or work burden concerns. Investigating how potentially modifiable factors are associated with both level and change may reduce the risk of ascribing undue importance to factors operating in older age.

